# Microstructure and Properties of Ni–Co Composite Cladding Coating on Mould Copper Plate

**DOI:** 10.3390/ma12172782

**Published:** 2019-08-29

**Authors:** Yu Liu, Ying Liu, Yali Gao, Chao Dong, Shoubo Wang

**Affiliations:** School of Mechanical Engineering, Northeast Electric Power University, Jilin 132012, China

**Keywords:** Ni–Co coating, laser cladding, mould, friction and wear, heat shock

## Abstract

In this study, a Ni–Co composite coating was fabricated successively on a copper substrate by laser, and the microstructure and properties of the composite coating were studied. Our results show that the elements from Ni and Co alloy powder were uniformly distributed in the binding region, which obtained a good metallurgical bonding with the substrate. The microhardness of Co-based coatings was 646 HV_0.1_, which was approximately 7.6 times greater than the hardness of Cu substrate. The Ni-based coating hardness was a little lower than that of the Co-based coating, which was approximately 7.0 times higher than the hardness of Cu substrate. The Co-based coating was comprised of reinforced phases, such as Co, Ni–Cr–Co–Mo, M_7_C_3_ and {Fe, Ni}. The wear volume of Co-based coatings was approximately 0.164 mm^3^ after the sliding wear test at 60 min, which was only 4% of the substrate’s wear volume. Meanwhile, the heat shock resistance experiment was carried out at 800 °C for 75 times before the Ni–Co composite coating was peeled off. It was shown that this Ni–Co composite coating had good wear resistance and high temperature properties, which is helpful to improve the service life of a mould copper plate. It not only saves the economic cost of steel plants, but also improves the quality of slabs and products.

## 1. Introduction

It is well known that mould is the key component of continuous casting, so it is usually called the “heart” of the continuous casting machine. Its service life will directly affect the slab quality and enterprise benefits. Due to the excellent thermal conductivity and corrosion resistance, copper and its alloy (Cu–Cr–Zr) are widely used as material for manufacturing mould copper plates. However, the copper plate is seriously worn and corroded during the actual production process because of high temperature and corrosion condition [1]. If the Cu element produced by the wear and friction enters into liquid steel, a large number of surface cracks will appear and the quality of the slab declines, which greatly limits the application of a mould copper plate [2]. Therefore, the wear resistance of a mould copper plate needs to be improved, which is helpful to prolong its service life and produce slab products of high quality. 

Some surface treatments, such as electroplating and thermal spraying, can be used to improve wear resistance of mould copper plates [3,4,5]. Although these methods help mould copper plates obtain better property, there are some disadvantages too. On the one hand, the interfacial binding region between coating and substrate has a lower strength due to mechanical bonding of electroplating and thermal spraying technology. Therefore, a mould copper plate often has the phenomenon that the electroplating coating is susceptible to being peeled off due to the high temperature and washing action of liquid steel [6]. On the other hand, low hardness of electroplating coatings is not helpful to obtain a long service life of a mould copper plate. Meanwhile, the electroplating technology is harmful to environment and will be eliminated gradually [7]. In the past decades, laser cladding is used more and more to fabricate coatings to improve the wear resistance and other properties [8,9,10,11,12,13]. Dehma et al. [14] fabricated a Ni-based cladding coating on Cu substrates. Yan et al. [15] prepared a Co-based/TiC/CaF_2_ self-lubricating composite coating on Cr–Zr–Cu mould. Due to the fact that performing laser cladding coating on mould copper plate has some difficulties, such as the high thermal conductivity and reflectivity, it is not easy to obtain cladding coating without defects. Therefore, researchers tried to make the first coating with one technology, and then produce a second coating with laser. Wang et al. [2] prepared a Ni coating with electroplating technology, and fabricated a Co coating with laser. Zhao et al. [16] prepared a stainless steel transition layer on copper substrates by friction stir welding, and a Ni-based alloy coating was made by laser cladding. Liu et al. [17] prepared a laser cladding coating of Ni-based alloy after plasma spraying. Zhou et al. [18] prepared a Cu–Fe alloy powder coating on a copper substrate, which improved wear resistance of mould copper plates. Although laser cladding on Cu substrates is studied by researchers, it is not easy to obtain a coating with good quality, and only using laser cladding to fabricate two layers has not been studied. In addition, if the electroplating method is used to make the first layer, the substrate and first layer are connected using a mechanical bond, which is still easily peeled off under high temperature work conditions. Therefore, in this study, a composite coating of the Ni-based transition layer and Co-based strengthened layer were fabricated on Cu–Cr–Zr substrates with laser. Then the morphology, microstructure, microhardness, wear resistance, and heat shock resistance of composite coatings were studied.

## 2. Materials and Methods

### 2.1. Materials 

**Figure 1:**
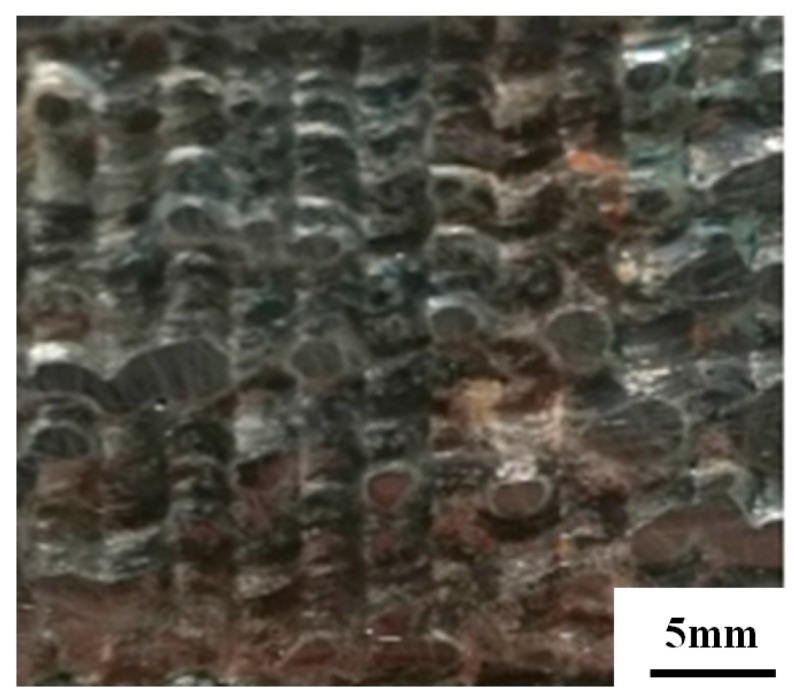
Macroscopic morphology of cladding coating.

The mould copper plate (Cu–Cr–Zr) was used as the substrate, and the size was 50 mm × 30 mm × 10 mm. The Ni-based (Ni60) and Co-based (Co42) alloy powders were used as cladding material and the composition is shown in Table 1. The alloy powder size was 200–300 meshes and laid on a copper substrate surface to 1 mm thickness. The device was a CO_2_ laser of a continuous mode and the parameters were as follows: laser power 1.6 kW, laser beam diameter 3.0 mm, scanning speed 100 mm/min, and overlapping ratio 30%. The experiments were carried out about 20 times. The two layers of cladding coating have the same parameters. The fabricated Ni–Co coating is shown in Figure 1.

### 2.2. Methods

The sample of laser cladding was cut into four pieces. The first piece was used for the scanning electron microscopy (SEM) experiments and energy dispersive spectrometer (EDS; 10 kV voltage). The second piece was used for the X-ray diffraction (XRD) experiments and hardness. The third piece was used for the friction and wear experiment. The last one was used for the heat and shock resistance experiment. After the sample was buffed and polished, an aqua regia solution was used to etch the cladding coating for 10 s. Then the sample was observed by optical microscopy and scanning election microscopy (SEM, QUANTA 200 FEI, Hillsboro, OR, USA). The elemental distribution of the binding region of Ni–Co and Ni–Cu were analyzed by EDS. The cladding layer phases were detected and analyzed by X-ray diffraction (XRD, TD-3500 Tongda Technology CO., Dandong, China). Hardness experiments for the Co-based coating, Ni-based coating, and copper substrate was carried out using a Vickers microhardness meter with a load of 100 gf at 10 s. The hardness was measured from the top of the Co-based coating to copper substrate, and each point was repeated five times at the same height. The friction and wear properties of the coating surface and substrate were tested using a reciprocating fatigue friction and wear tester (MGW-02, Jinan Yihua Tribology Testing Technology CO., Jinan, China), with tip force of 10 N and scratch speed 100 r/min. This experiment was repeated three times, and average volume loss was calculated. The experiment of heat shock was also carried out to obtain the high temperature property of cladding coating. The sample was heated to 800 °C and kept for 5 min, then put into water for cooling. This process was carried out until the coating was peeled off. 

## 3. Results and Discussion

### 3.1. Phases

**Figure 2:**
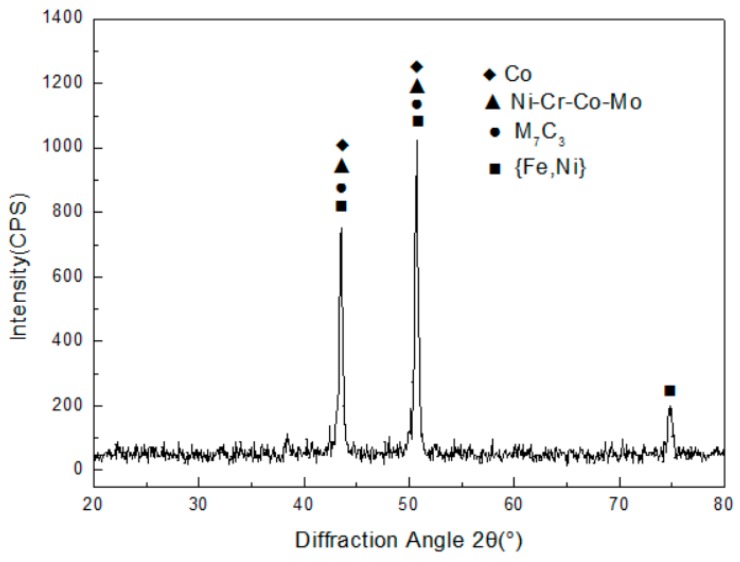
X-ray diffraction result of Co-based cladding coating.

The X-ray diffraction pattern of Co-based coatings is shown in Figure 2. The Co and Ni–Cr–Co–Mo solid solution were the predominant phases, and the solid solution of M_7_C_3_ and Fe–Ni were also found in the surface of cladding coating. Here, M is Cr and Fe elements. Although there was no Ni element in the Co42 alloy power in Table 1, the first Ni-based coating was melted when the Co-based cladding coating was fabricated. The solid solution and carbides, such as Ni-Cr-Co-Mo, M_7_C_3_ and Fe–Ni, were composed to act as the reinforced phases of cladding coating.

### 3.2. Microstructure and Binding Region Analysis 

**Figure 3:**
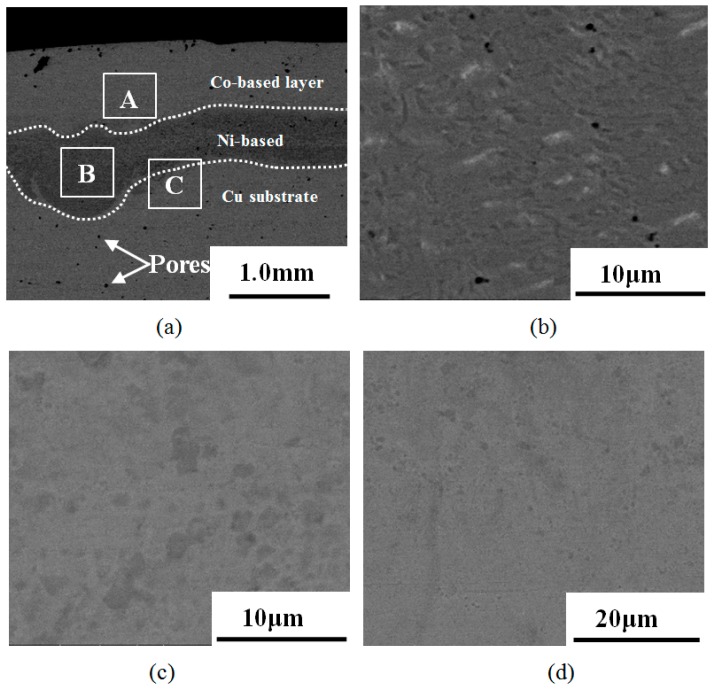
(**a**) Two layers coating; (**b**) Co-based layer; (**c**) Ni-based layer; (**d**) Ni-Cu zone cross-section microstructure morphologies.

Figure 3 shows the cross-sectional macroscopic morphology of the composite coating after laser cladding. The whole morphology of composite coating is shown in Figure 3a. It can be seen that Co-based coating, Ni-based coating, and copper substrate have obvious boundary. There are also some pores in the coating layers and substrate. The size ranged from 1 µm to 150 µm. The formation of pores is usually due to the overheating of the molten pool, gas entrapment, surface impurities, etc. [19]. These pores in the coating decrease the service life of a mould copper plate. It can be seen that Cu substrate have the most pores. The reason is that copper easily captures gas in the air at high temperatures, which cannot escape from the molten pool timely due to the rapid cooling rate. It can also be seen that the pores of Co-based coatings were more than those in the Ni-based coating, which is caused by pore floating during the solidification process. Therefore, there are more pores in the Co-based coating. Figure 3b represents the microstructure of region A in Co-based coatings. The coating is composed of disordered dendrites and plane crystals. The main reason is that the coating’s surface has a high cooling rate and forms some fine dendrites. With the formation of surface grains, the temperature gradient decreases, and the cooling rate is lower. Then dendrites are gradually formed. Figure 3c shows the microstructure of region B in Ni-based layer, which is composed of gray dendrites and cellular dendrites. The binding region C of Ni–Cu is shown in Figure 3d. It shows that Ni-based alloy powder obtains a metallurgical binding region without defects.

**Figure 4:**
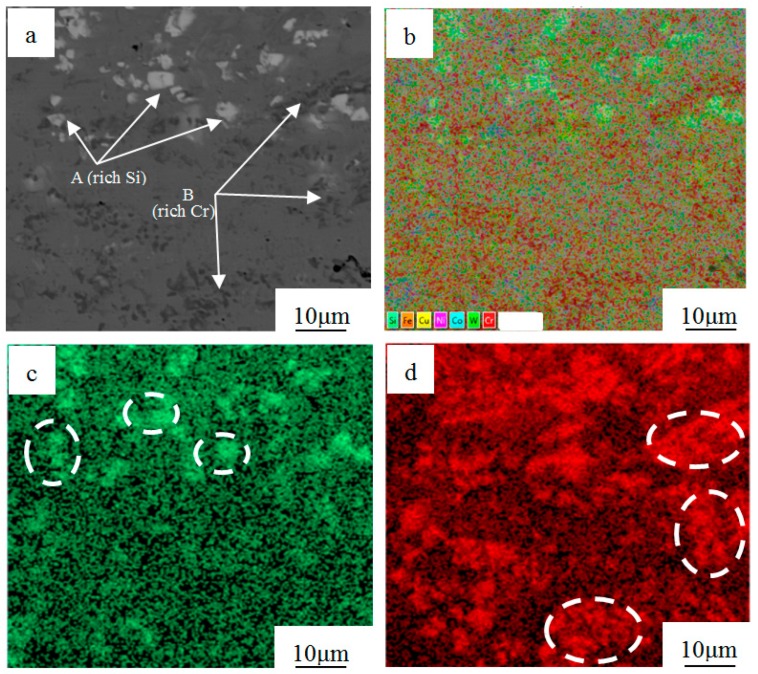
Scanning electron microscopy image (**a**) and distribution of all elements (**b**), Si (**c**), Cr (**d**) of the Ni–Co binding region.

Figure 4 depicts a SEM micrograph and the distribution of Si, Cr, and Ni elements of the Ni–Co binding region. From the SEM micrograph of Figure 4a, it can be seen that there are some obvious regions like A and B. The whole mass fraction of each element is shown in Table 2. The amount of Cu, Ni, Cr, and Co elements are relatively large and distributed uniformly around the binding region as shown in Figure 4b, forming a solid solution. This is helpful for obtaining good metallurgical bonding of Ni–Co coatings. The data further show that the marked region A is rich in Si and region B is rich in Cr (Figure 4c,d), which form SiC and Cr_7_C_3_ with carbon. 

**Figure 5:**
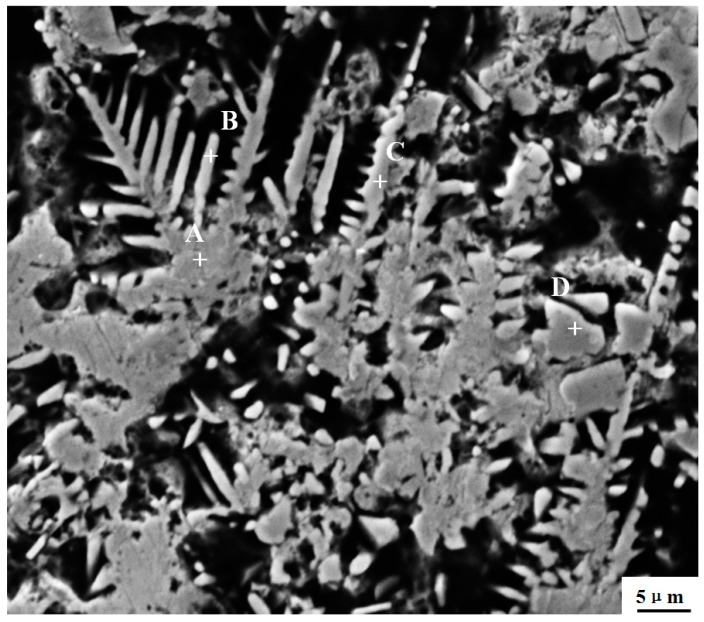
SEM image of the Ni–Co binding region microstructure.

Figure 5 shows SEM image of the microstructure of Ni–Co binding region, showing cellular dendrites. The EDS spot analyses were used to obtain the chemical compositions of the phases with different morphologies. The result is shown in Table 3. Our data show that the whole Ni–Co transition coating is rich in Cr, Ni, and C accompanied with some dissolved elements such as Fe and B. The area A is rich in Ni, Cu, and C, which forms a Ni-based solid solution. Areas B and C are rich in Cr, C, and Ni, which form carbides and Ni-based solutions. Area D has some Cr and B compounds. It can be concluded that Cr and its compound are the main phases in Ni–Co coatings.

### 3.3. Microhardness 

**Figure 6:**
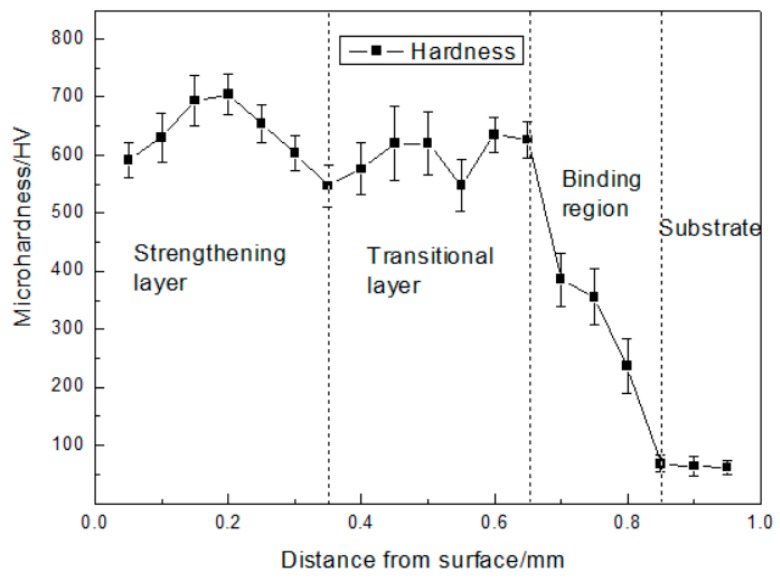
Microhardness variation of the cladding coating’s cross-section.

Figure 6 depicts the hardness variation of the Co-based strengthening region, Ni-based transition region, and copper substrate. The thickness of Co-based cladding coating was about 0.35 mm. The average hardness of Co-based coating was 646 HV_0.1_, which is ~7.6 times greater than the hardness of the Cu substrate. It is helpful to improve the wear resistance of the composite coating. The thickness of the Ni-based cladding coating was ~0.3 mm. The Ni-based coating hardness was 596 HV_0.1_, lower than that of Co-based coatings, which is ~7.0 times greater than the hardness of the Cu substrate. The binding region’s thickness was ~0.2 mm and the average hardness was 326 HV_0.1_. The hardness between the Ni-based coating and substrate is decreased gradually, so that the whole cladding coating can be well bonded to substrate. 

### 3.4. Wear Resistance 

**Figure 7:**
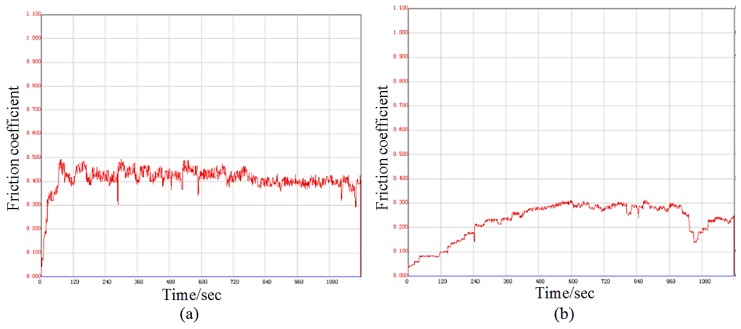
Friction and wear curve for the copper substrate (**a**) and Ni–Co coating (**b**).

**Figure 8:**
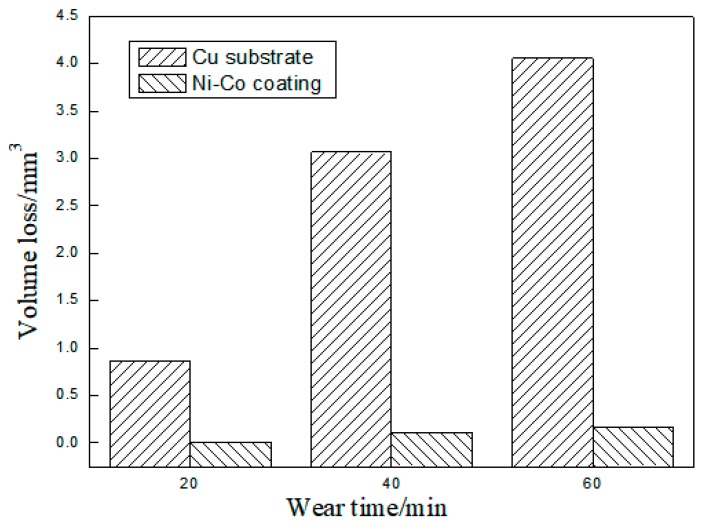
Wear volume of the copper substrate and Ni–Co coating along with different times.

The friction coefficient of the copper substrate and Ni–Co composite coating is shown in Figure 7. The friction coefficient of the copper substrate was between 0.4 and 0.5. The friction coefficient of the cladding coating rose initially and then stabilized around 0.3. Then the friction and wear tests of the copper substrate and Ni–Co composite coating was carried out. Wear scar width was measured in order to calculate the wear volume of substrate and Ni–Co composite coating by Equation (1) [20]. Figure 8 shows wear volume of the copper substrate and Ni–Co coating along with different times. The wear volume of copper substrate and Ni–Co coating is 0.83 mm^3^ and 0.008 mm^3^ at 20 min, which is only 1% the volume of the Cu substrate. The wear volume of the copper substrate was 4.05 mm^3^ when the wear time reached 60 min, while the wear volume of Ni–Co coating was 0.164 mm^3^, which is approximately 4% the Cu substrate’s volume. These results demonstrate that the Ni–Co coating has a better wear resistance and lower wear volume than the Cu substrate.

(1) $$V=\frac{1}{2}R^{2}L\left[2\arcsin\left(\frac{B}{2R}\right)-\sin2\arcsin\left(\frac{B}{2R}\right)\right]$$

where V is the wear volume; R is the radius of the grinding ball, 2.5 mm; L is the wear scar length; B is the wear scar width. 

### 3.5. Heat Shock Resistance 

**Figure 9:**
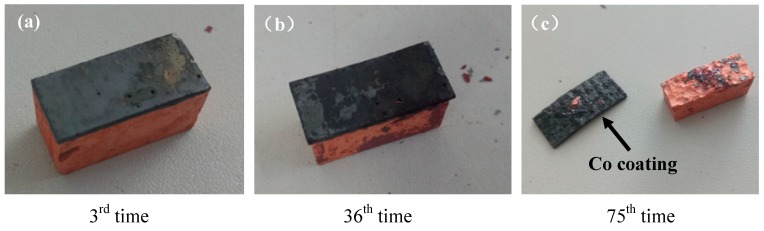
Macro morphology of the sample after the 3^rd^ (**a**), 36^th^ (**b**), and 75^th^ (**c**) experiments of heat shock resistance.

Figure 9 represents the macro-morphology of the sample after the 3rd, 36th, and 75th heat shock resistance experiment. The results show that the cladding coating has an oxidation phenomenon after the 3rd experiment, but there is no obvious deformation. However, after the 36th experiment, there was a severe oxidation phenomenon on the surface of the coating and substrate as shown in Figure 9b. It can be seen that the volume of Cu substrate becomes smaller due to the off of oxides, while the Ni–Co coating has less oxide which makes the volume loss of cladding coating less. After the 75th experiment, the Ni–Co cladding coating was peeled off from the Cu substrate due to the different shrinkage stresses caused by heating and cooling, thereby breaking the Ni coating layer. The Cu substrate and cladding coating were smaller than the primary ones, and shrinkage of the Cu substrate was more obvious than that of the Ni–Co cladding coating. Therefore, the Ni–Co coating is bent to the direction of substrate. As shown in Figure 9c, the peeled layer is the Co coating, which also keeps a whole shape without any cracks. This illustrates that the Co coating has a very good high temperature property. 

## 4. Conclusions

(1) A Ni–Co composite coating was fabricated on a copper substrate using laser. The surface of the Co-based coating has some reinforced phases, such as Co, Ni–Cr–Co–Mo, M_7_C_3_ and {Fe, Ni}. All metal elements are uniformly distributed between the Ni–Co binding region, which forms a good metallurgical bonding.
(2) The average hardness of the Co-based coating is 646 HV_0.1_, which is 7.6 times greater than the hardness of the Cu substrate. Furthermore, the hardness of Ni-based coating hardness is 596 HV_0.1_, which is approximately 7.0 times greater than that of the Cu substrate.
(3) The wear volume of the Co-based coating is 0.164 mm^3^ at 60 min, which is only 4% that of the Cu substrate’s wear volume. Therefore, this composite Ni–Co coating has a better wear resistance and lower wear volume.
(4) The Ni–Co coating is peeled off after the 75th experiment of heat shock resistance at 800 °C. There is no defect during this process. This coating has a very good high temperature property.

## Figures and Tables

**Table 1:** Elemental composition (wt.%) of Ni60 and Co42 alloy powder.

Powder		Element (wt.%)							
	C	Cr	Si	B	Fe	W	Mo	Co	Ni
Ni60	0.45	13.5	4.0	2.5	15	-	-	10.5	Bal.
Co42	1.4	32	2.0	1.2	2.0	6.0	0.8	Bal.	-

**Table 2:** Mass fraction of each element at the binding region of the Ni–Co coating (wt.%).

Cu	Ni	Cr	Co	B	W	C	Fe	Si	Mo
29. 3	28.0	15.8	9.1	6.2	5.3	2.2	2.0	1.7	0.4

**Table 3:** Chemical composition of four areas in the Ni–Co transition coating (wt.%).

Areas	Cr	Ni	Fe	W	B	C	Cu	Si
A	6.1	58.0	4.9	-	-	8.0	19.8	3.2
B	62.3	7.3	3.2	-	-	26.9	-	0.3
C	60.5	15.9	3.8	-	-	17.9	1.9	-
D	84.1	3.2	1.8	1.7	9.0	-	-	-
